# Bleaching drives collapse in reef carbonate budgets and reef growth potential on southern Maldives reefs

**DOI:** 10.1038/srep40581

**Published:** 2017-01-13

**Authors:** C. T. Perry, K. M. Morgan

**Affiliations:** 1 Department of Geography, College of Life and Environmental Sciences, University of Exeter, Exeter EX4 4RJ, United Kingdom

## Abstract

Sea-surface temperature (SST) warming events, which are projected to increase in frequency and intensity with climate change, represent major threats to coral reefs. How these events impact reef carbonate budgets, and thus the capacity of reefs to sustain vertical growth under rising sea levels, remains poorly quantified. Here we quantify the magnitude of changes that followed the ENSO-induced SST warming that affected the Indian Ocean region in mid-2016. Resultant coral bleaching caused an average 75% reduction in coral cover (present mean 6.2%). Most critically we report major declines in shallow fore-reef carbonate budgets, these shifting from strongly net positive (mean 5.92 G, where G = kg CaCO_3_ m^−2^ yr^−1^) to strongly net negative (mean −2.96 G). These changes have driven major reductions in reef growth potential, which have declined from an average 4.2 to −0.4 mm yr^−1^. Thus these shallow fore-reef habitats are now in a phase of net erosion. Based on past bleaching recovery trajectories, and predicted increases in bleaching frequency, we predict a prolonged period of suppressed budget and reef growth states. This will limit reef capacity to track IPCC projections of sea-level rise, thus limiting the natural breakwater capacity of these reefs and threatening reef island stability.

Coral reefs support a wealth of ecosystem goods and services that extend across the provisioning of food resources, through cultural and tourism benefits, to those associated with shoreline protection^1^,^2^,^3^. Climate change poses a major threat to the capacity of reefs to sustain these functional roles. Warming sea waters, ocean acidification and rising sea levels all have the potential to impact reefs across a range of spatial and temporal scales, regardless of local management or degree of geographic isolation^4^,^5^,^6^,^7^. Critical in the context of ecosystem service sustainability will be the extent to which these various climate change related stressors, acting either independently or in tandem with other direct human-induced disturbances (such as long-term resource over-extraction), will modify reefs both ecologically and in terms of their carbonate budgets^8^,^9^,^10^. A reefs carbonate budget represents the balance between the rate at which carbonate is produced by corals, coralline algal and other carbonate producing processes, less the rate at which carbonate is removed by either biological erosion (‘bioerosion’), physical processes, or chemical dissolution^8^. Such budget state measures have recently been identified as a key metric for assessing reef “health”^11^, but in terms of ecosystem service provisioning the budget state of a reef is of particular relevance because of the direct influence exerted on the capacity of reefs to maintain both their physical 3-dimensional structures and their vertical growth potential^8^. Shifts towards net negative budgets may thus lead to reef structural collapse, loss of reef growth potential, and diminished ecosystem service provisioning^9^,^10^,^12^.

Negative budget trajectories have already become evident across regions such as the Caribbean, where the effects of prolonged and systematic ecological decline, caused both by direct human disturbances and a suite of coral diseases, have resulted in steady transitions to states of low coral carbonate production^13^,^14^, reduced carbonate budgets and diminished reef growth potential^12^. Thus the capacity of most Caribbean reefs to keep pace with future sea-level rise is likely to be extremely limited^12^. What is more poorly understood, however, are how individual events or disturbances may modify reef carbonate budgets. In this context, and in relation to predictions of ever more frequent and severe sea-surface temperature warming^15^,^16^,^17^, the impacts of coral bleaching-driven mortality events are especially important. This is because the ecological impacts of bleaching are near-instantaneous and can be severe. Such events thus have the capacity to also drive very rapid, and potentially severe, declines in carbonate budgets^18^ and in resultant reef growth potential. Here we quantify the impacts of the most recent large-scale sea-surface temperature (SST) anomaly event in the central Indian Ocean, driven by the strong ENSO-induced warming of 2016. Specifically, we quantify the impacts on atoll interior reefs in the southern Maldivian atoll of Gaafu Dhaalu (Fig. 1A), a location where other common drivers of major reef degradation exert minimal influence (fishing pressure on these reefs is relatively low, and immediate point sources of nutrient input absent). Satellite SST anomaly data indicates that strong warming in this region started in late March 2016 (Fig. 1B) and persisted at levels above the regional bleaching threshold of ~30.9°C through until mid-May 2016 (Fig. 1B–D), the bleaching threshold being defined as the point where SST is 1 °C warmer than the highest monthly mean temperature (NOAA, 2016). Here we use pre- (January 2016) and post-warming (September 2016) measured rates of both gross carbonate production and bioerosion from shallow fore-reef habitats (2 m depth) on five reefs (Mahutigala, Kandahalagala, Kafigahlaa, Kodehutigalaa and Kadumaigala) to determine their net biological carbonate budgets (G, where G = kg CaCO_3_ m^−2^ yr^−1^). We use these data to address two specific questions: 1) what impact did the 2016 warming event have on the ecological composition and carbonate budgets of the shallow fore-reef (2 m depth) habitats, and how consistent were the responses across these reefs?; and 2) what have been the resultant impacts on reef growth potential?

## Results

**Figure 1:**
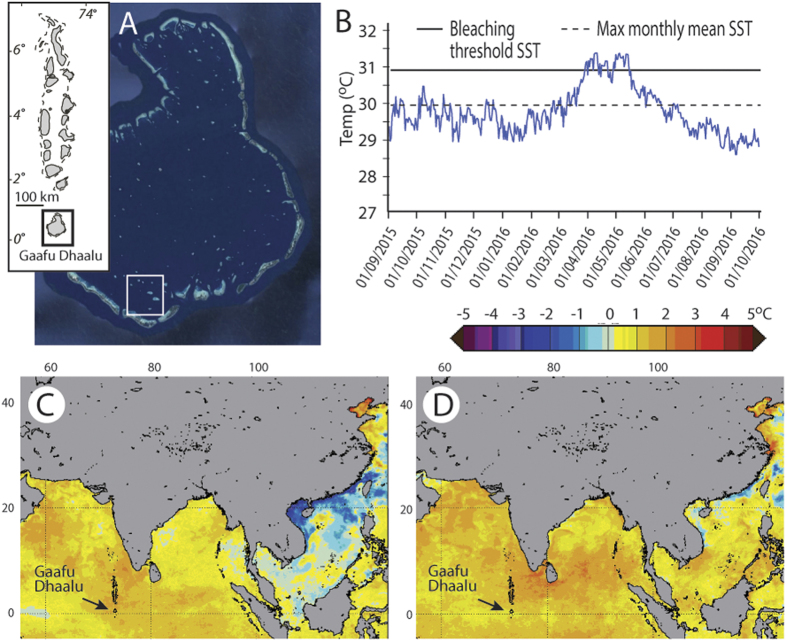
Study location and SST anomaly data. (**A**) Location of Gaafu Dhaalu atoll (inset map) in the southern Maldives, and the location of the study sites in the southern part of the atoll (white boxed area). Inset map (traced using Adobe Illustrator Version CS5) and satellite image from Google Earth Imagery (Map data: Google, Landsat 2016); (**B**) Time-series data showing satellite-derived sea-surface temperature (SST) data for the Maldives (01/09/15 to 31/09/16). Daily data were extracted and replotted from the NOAA Coral Reef Watch site (http://coralreefwatch.noaa.gov/vs/gauges/maldives.php), accessed 03/10/16; (**C,D**) Maps showing satellite derived SST anomaly data for the Indian Ocean; (**C**) 1^st^ March 2016, (**D**) 1^st^ May 2016. Plots derived from NOAA Coral Reef Watch. 2016, updated daily. NOAA Coral Reef Watch Daily Global 5-km Satellite Virtual Station Time Series Data for Indian Ocean, Mar. 1, 2016 and May 1, 2016. College Park, Maryland, USA: NOAA Coral Reef Watch. Data set accessed 2016-10-03 at http://coralreefwatch.noaa.gov/vs/index.php.

**Figure 2:**
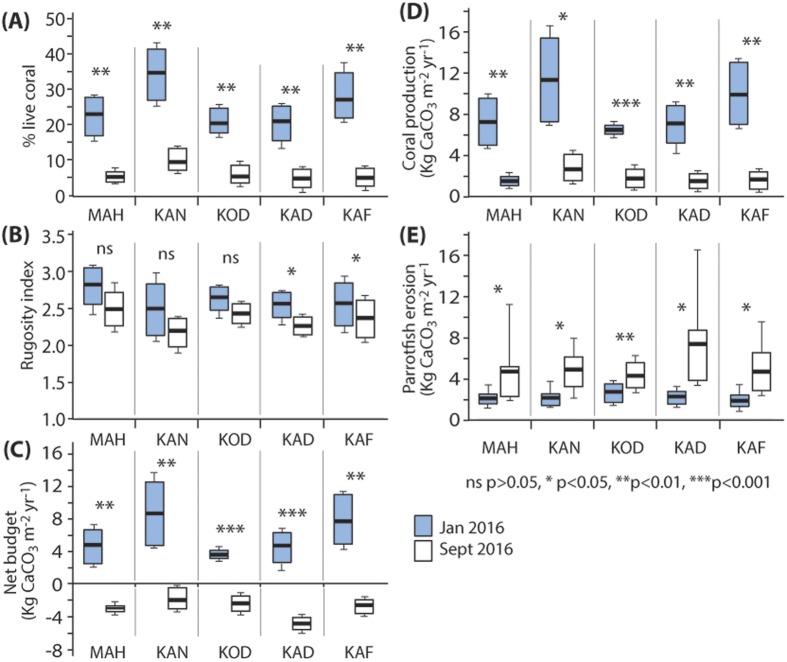
Comparisons between key ecological, structural and carbonate budget metrics across reef sites in Gaafu Dhaalu atoll, Maldives between January 2016 and September 2016. Box (median and 50% quartile) and whisker (95% quantile) plots showing differences in; (**A**) coral cover, (**B**) rugosity, (**C**) net budget, (**D**) coral production, and (**E**) parrotfish erosion. MAH – Mahutigala, KAN – Kandahalagala, KOD – Kodehutigalaa, KAD – Kadumaigala, KAF – Kafigahlaa.

The extensive coral mortality that occurred between January 2016 and September 2016 at all our study sites can be linked with a high degree of certainty to the strong SST warming that occurred across the central Indian Ocean region in mid-2016 (Fig. 1B,C). Coral cover (measured as cover of the 3-dimensional surface of the reef) declined significantly between January 2016 (across site mean: 25.6 ± 5.8%; range: 20.7 to 34.9%; Supplementary Table 1) and September 2016 (mean: 6.3 ± 1.9%; range: 5.2 to 9.7%) (p < 0.01; Supplementary Table 2), and at each site the decline exceeded 70% (mean 75.6 ± 3.2%; Supplementary Table 1). Much of this decline was driven by widespread mortality of branching and tabular *Acropora* spp. (Supplementary Figure 1), the relative abundance of which has declined significantly at all sites (p < 0.01; Supplementary Table 2), and with average % abundance of *Acropora* spp. declining by almost an order of magnitude across sites (Jan 2016: 14.72 ± 5.04%, Sept 2016: 1.19 ± 0.46). Observational data indicates that extensive *Acropora* spp. mortality occurred to depths of ~5–6 m at all sites. Whilst many massive and sub-massive morphology taxa (including *Porites* spp.) survived the bleaching (albeit with some partial mortality evident), fundamental changes to the ecology of these shallow fore-reef habitats have occurred. As of early September 2016 most dead *Acropora* colonies remain in living position (Supplementary Figure 1), but declines in substrate rugosity (mean decline 10.1 ± 2.0%) are already becoming evident (Jan 2016 mean 2.6 ± 0.3, range: 2.5 to 2.8; Sept 2016 mean 2.3 ± 0.1%, range: 2.2 to 2.3%; Fig. 2B), although significant declines have only occurred at Kadumaigala and Kafigahlaa (p < 0.05, Supplementary Table 2).

Whilst the structural complexity of these shallow fore-reef habitat sites is thus far relatively unchanged, widespread coral mortality has resulted in a very significant decline (mean decline 157.5 ± 30.9%) in the net carbonate budgets at all five sites (p < 0.05; Supplementary Table 2), shifting from strongly net positive (Jan 2016, mean 5.92 ± 2.2 G, range: 3.6 to 8.6G; Fig. 2C) to strongly net negative (Sept 2016 mean −2.96 ± 1.06 G, range: −1.9 to −4.7G; Fig. 2C). These substantial carbonate budget declines reflect two interacting variables. Firstly, measured rates of coral carbonate production have declined significantly at all sites (p < 0.05, Supplementary Table 2) (Jan 2016 mean 8.43 ± 2.08 G, range: 6.4 to 11.3 G; Sept 2016 mean 1.83 ± 0.47 G, range: 1.5 to 2.6 G), an average decline of 78.0 ± 3.1% (Fig. 2D). At the same time, measured rates of parrotfish bioerosion have increased substantially (+139.5 ± 59.7%; Fig. 2E). These increases are significant at all sites (p < 0.05, Supplementary Table 2; Jan 2016 mean 1.76 ± 0.24 G, range: 1.4 to 2.1 G; Sept 2016 mean 4.15 ± 0.99 G, range: 3.4 to 5.8 G).

**Figure 3:**
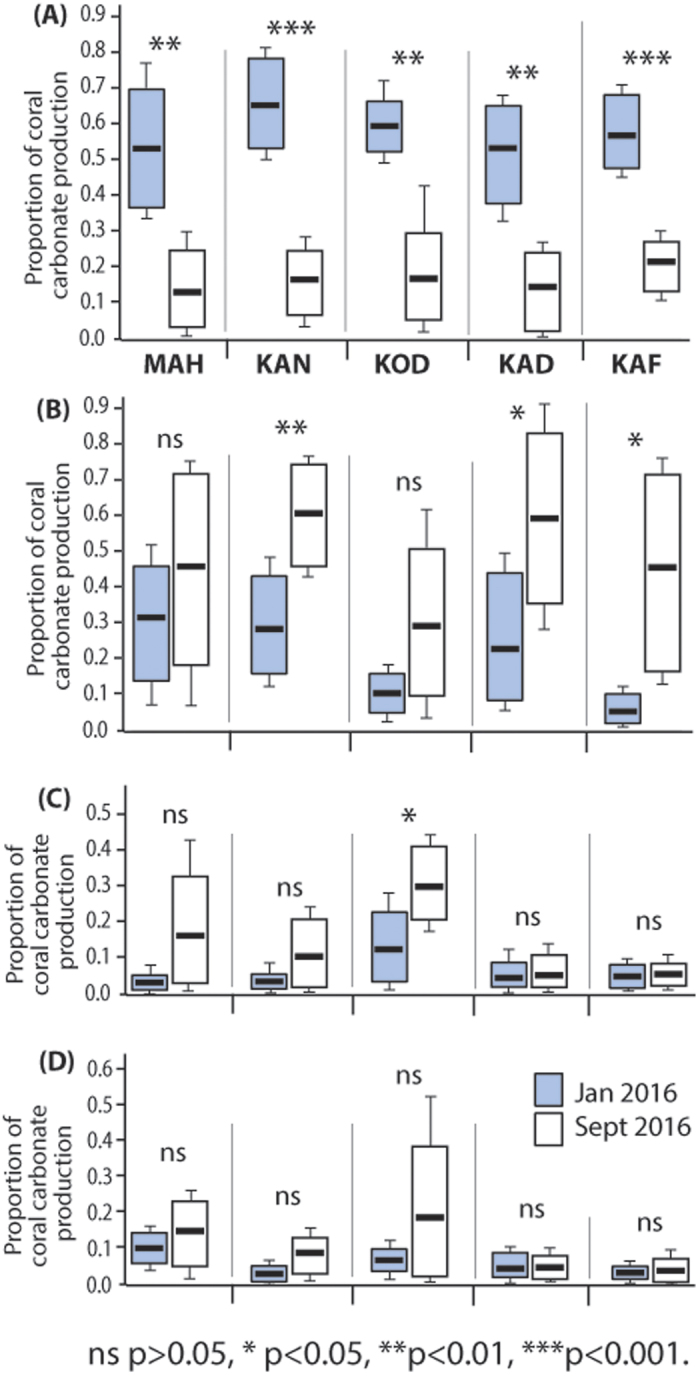
Proportional contributions to coral carbonate production rates (kg CaCO_3_ m^−2^ yr^−1^) across reef sites in Gaafu Dhaalu atoll, Maldives between January 2016 and September 2016. Box (median and 50% quantile) and whisker (95% quartile) plots showing differences in; (**A**) *Acropora* (all branched and tabular species), (**B**) Massive and sub-massive taxa (including *Porites*), (**C**) Non-*Acropora* branched taxa, including *Pocillopora* spp., and (**D**) Encrusting taxa. MAH – Mahutigala, KAN – Kandahalagala, KOD – Kodehutigalaa, KAD – Kadumaigala, KAF – Kafigahlaa.

Our data also indicate marked post-warming shifts in the proportional contributions made to coral carbonate production rates by different groups of coral taxa. Pre-coral bleaching (Jan 2016) coral carbonate production was dominated by branching, corymbose and tabular species of *Acropora* (mainly *A. cytherea, A. digitifera, A. muricata, A. lamarcki*), which collectively accounted for between 52 ± 15.3% and 65.2 ± 13.7% of coral carbonate production (Fig. 3A). Post-bleaching, the contribution of *Acropora* spp. declined on average by 70.9 ± 5.6%, and was significant at all sites (p < 0.01, Supplementary Table 2) (Jan 2016 mean 57.2 ± 13.7%, range: 30.0 to 81.0%; Sept 2016 mean 16.6 ± 12.2%, range: 0–32.2%). In contrast, proportional contributions by other more resilient massive and sub-massive taxa (*Porites* spp., *Favia* spp.) more than doubled (Jan 2016 mean 19.7 ± 16.8%, range: 1.0 to 54.0%; Sept 2016 mean 47.6 ± 27.8%, range: 0–95.0%), and was significant at all sites (p < 0.05, Supplementary Table 1), except Mahutigala (p = 0.42) and Kodehutigalaa (p = 0.13) (Fig. 3B). Proportional contributions to coral G by other non-*Acropora* branching (mainly *Pocillopora* spp.) taxa also increased (Jan 2016, mean 4.5 ± 0.6%, range: 0 to 28.0%; Sept 2016, mean 12.2 ± 14.1%, range: 0–48.0%) (Fig. 3C), as did contributions from encrusting taxa (Jan 2016, mean 5.6 ± 4.2%, range: 1.0 to 16.0%; Sept 2016, mean 10.0 ± 12.9%, range: 1.0–61.0%) (Fig. 3D), but overall contributions to coral G by these taxa remain relatively small, and measured increases are non-significant (Supplementary Table 2).

**Figure 4:**
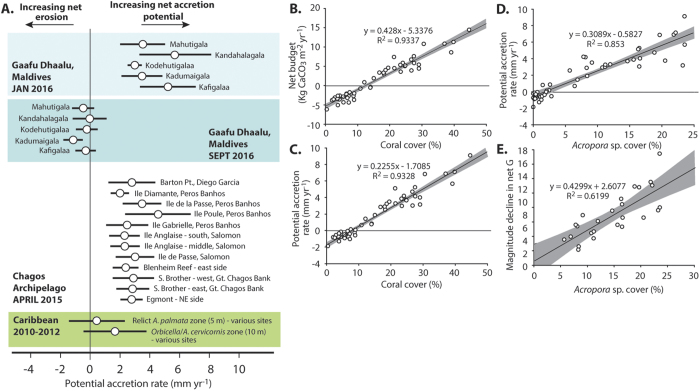
Coral reef accretion rates. (**A**) Changes in rates of reef accretion (mean mm yr^−1^ +/−1 standard deviation) between atoll interior reef sites in Gaafu Dhaalu atoll, Maldives between January 2016 and September 2016. All sites are at 2 m depth on the fore-reef slopes. Comparative data from *Acropora*-dominated sites around the Chagos Archipelago, central Indian Ocean (all 9 m depth) (in 23), and from the Caribbean (in 12), and recalculated based on a conservative assumption of 20% of framework being removed by physical processes. (**B–D**) The linear regression and 95% confidence intervals for the relationships between: (**B**) Coral cover and net carbonate production (**G**); (**C**) Coral cover and reef accretion potential (mm yr^−1^); (**D**) *Acropora* spp. cover and reef accretion potential (mm yr^−1^); and (**E**) *Acropora* spp. cover pre-bleaching (Jan 2016) and the magnitude of the subsequent reduction in the net carbonate budget (**G**).

Based on the carbonate budgets measured around the five study sites in January 2016, calculated rates of vertical reef growth within the shallow fore-reef habitats averaged 4.2 mm yr^−1^ (range: 1.4 to 9.1 mm yr^−1^; Fig. 4A). Our data show, however, that following the warming event of mid-2016, very significant reductions in reef growth potential occurred across all sites (p < 0.001; Supplementary Table 2), with mean rates at all sites now net negative i.e., net erosional (average −0.43 mm yr^−1^, range: 0.15 to −1.88 mm yr^−1^) (Fig. 4A). This points to not only a rapid loss of growth potential, but also the likelihood that the structural complexity of these reefs, which in the immediate aftermath of the event has only marginally declined (see Fig. 2B), will progressively be diminished.

## Discussion

The major and prolonged period of elevated SST that caused coral bleaching in the Maldives in 2016 resulted from the recent strong El Niño that originally begun forming in the central Pacific region in June 2014^19^. This mid-2014 warming event ultimately is thought to have initiated the much larger scale warming that spread across the world’s oceans, and caused widespread coral bleaching, through late 2015 and mid-2016. Indeed, such has been the scale of resultant bleaching that the event has been formally designated as the “Third global coral bleaching event”^20^. Peak temperatures in the Maldives occurred between March and May 2016 (Fig. 1), and our data from the southern Maldives suggests that this resulted in very significant ecological changes. Coral cover declined by an average 75% and the overall impact of this on the carbonate budgets of the shallow fore-reef habitats on these reefs has been profound. Carbonate budgets have reduced by an average of 157%, and all sites now have net negative budgets. Critically, in terms of the wider geographic relevance of these findings, we note that our coral cover decline data are consistent with findings from rapid assessments undertaken during mid-2016 across the wider Maldives, which also indicated between 60 and 90% coral mortality on shallow reefs^21^. This suggests that geographically widespread reductions in reef carbonate budgets and thus in reef growth potential, of the magnitude we report here, are highly likely to have occurred.

A key driver of these declines has been the mass mortality of branched and tabular *Acropora* spp., the cover of which has declined by an average 91%. This not only has major budget implications, but also major ecological implications because of the high habitat complexity and diversity such corals provide within these shallow fore-reef habitats^22^. Interestingly, we note that mortality of another important shallow water fore-reef taxa (*Pocillopora*), which is often badly impacted by bleaching^6^,^22^, suffered variable levels of mortality. Some colonies clearly survived this event, whilst others show evidence of only partial mortality, but the proportional contribution of this taxa to coral carbonate production increased slightly at most sites (Fig. 3). However, despite these taxa specific responses, rates of overall coral carbonate production declined markedly (by more than 300%), from a pre-event average of 8.4 G to a post-bleaching rate of 1.8 G. We also note that rates of bioerosion by parrotfish increased significantly across sites (+139%) in the pre- and post-bleaching interval. This, we hypothesise, must reflect an increase in the exploitation of newly available, filamentous algal covered dead coral substrate by parrotfish at these sites. Thus the carbonate budgets of these reefs have been strongly impacted by both a rapid decline in coral carbonate production and by an increase in substrate bioerosion rates.

The net effect of these budget changes has been to drive very substantial declines in shallow fore-reef growth potential, which have declined from an average 4.2 mm yr^−1^ to −0.4 mm yr^−1^. We note that pre-bleaching rates are similar to those measured at *Acropora*-dominated sites around the remote Chagos Archipelago (central Indian Ocean) in mid-2015 (mean: 2.9 mm yr^−1^, range: 0.9 to 6.3 mm yr^−1^; Fig. 4A)^23^, albeit with the Chagos data being derived from slightly deeper water (9 m depth) fore-reef habitats, and with communities their dominated by tabular, rather than branched *Acropora* species. Notwithstanding these inter-site differences, the similarities in site average rates are clear, and suggest that potential growth rates in the order of ~3 to 4 mm yr^−1^ might be considered realistic for many “healthy”, shallow-water (<10 m depth) *Acropora*–dominated reefs in the central Indian Ocean, a rate supported by metadata analyses of shallow water Indian Ocean accretion rates^24^.

A key implication of the major post-bleaching changes we report is that, at least at present, the shallow fore-reef habitats on these Maldivian reefs have shifted from states defined by strong growth potential to a situation dominated by net framework erosion and breakdown. This has potentially very significant implications in terms of the capacity of these reefs to continue acting as breakwater structures for the reef islands that they support. In the short-term (the next few years) the breakdown and loss of shallow fore-reef surface structural complexity will likely reduce the effectiveness of these reefs to reduce wave energy propagation across the reef flats^25^,^26^. This is perhaps an especially important issue in these settings because of the role that the surrounding reefs play in modulating wave energy regimes around islands^27^ and thus the short-term morphodynamic responses of the islands they underpin^28^,^29^. However, in the medium-term (decades), any continuation of the current states of low (negative) budget states will also progressively impinge upon the capacity of the reefs to match any increases in sea-level. Given the rapid increases in wave energy propagation that follow the increased submergence of reefs^3^,^30^ the impacts for island stability in the region may thus be significant.

As at other sites where relationships between coral cover and carbonate budgets have been examined^14^,^23^, we note a strong positive correlation between both net carbonate production rates and reef accretion (Fig. 4B,C). In our datasets the average coral cover threshold for the maintenance of positive carbonate budgets is around 12% (Fig. 4B), and for net positive accretion potential around 8% (Fig. 4C). These thresholds are broadly comparable to those measured from multiple sites around Chagos further south in the Indian Ocean^23^, and thus appear to be consistent not only across the Indian Ocean region, but are also comparable to those derived for the Caribbean region^12^. It is important to emphasise, however, that these % cover data reflect that measured as a function of the true 3 dimensional cover of the reefs, and thus equate to higher % cover thresholds where data is collected using linear point or photo/video quadrat type methods.

In the present study we also note that the abundance of fast growing *Acropora* species is an especially important control on budgets and accretion potential. *Acropora* cover and accretion rate are strongly correlated (Fig. 4D), and there is a strong positive relationship between the % cover of *Acropora* on these Maldivian reefs, and the magnitude of decline in their carbonate budgets post-bleaching (Fig. 4E). In other words, reefs with the highest initial cover of *Acropora* experienced the largest magnitudes of decline in carbonate budgets and thus in accretion potential. This has important implications that relate to on-going discussions around ecological “winners” and “losers” under severe climate stress^22^,^31^,^32^,^33^. As we move into a period where major SST anomaly events are predicted to increase in frequency and intensity^34^, the extent to which reefs are colonised by either “winner” or “loser” taxa will have direct relevance for predicting the long-term capacity of reefs to sustain periods of strong positive budgets and growth potential. For example, communities dominated by branched, corymbose and/or tabular *Acropora* species (depending on setting and energy exposure) typically dominate in shallow-water Indian Ocean reef habitats. These same species also drive high rates of coral framework production and result in high potential accretion rates (even factoring for substantial amounts of annual framework export due to physical breakage and export; ref. 23). However, it also well established that these same species are amongst the most vulnerable to SST-driven stress^32^, and this has two key implications. Firstly, it is likely that it is the most “pristine” reefs (i.e., those with high branched/tabular *Acropora* cover) which will be the most susceptible to future bleaching events, and thus the most susceptible to future rapid declines in carbonate production and reef accretion. Secondly, it must also be assumed that if bleaching events become more severe and frequent then the capacity (and timescales necessary for) the recovery of *Acropora* communities will progressively diminish, potentially to the point that even the most remote and protected reefs will struggle to recover from disturbances in terms of their budgets and growth potential.

An important issue now will therefore be whether and when these reefs may recover, both ecologically and in terms of their carbonate budgets. Evidence from other sites in the region which were strongly impacted by the 1997/98 bleaching event suggest some grounds for optimism, especially where the reefs are relatively remote or isolated from high levels of direct human disturbance. For example, the reefs around the remote Chagos archipelago (to the south of the Maldives) showed an impressive capacity for recovery from the 1997/98 mass coral bleaching event. Coral cover on most reefs around Chagos had recovered to pre-bleaching levels by around 2010^35^ i.e., within about 12 years, and those reefs (as of May 2015) were characterised by strongly positive carbonate budgets and high reef accretion potential^23^. Many reefs in the Maldives were also severely impacted by the 1997/98 event^22^,^36^, but subsequent monitoring across a number of atolls showed that whilst encrusting and massive taxa dominated the early post-bleaching communities, it is now the case, as at our study sites, that these had been replaced by species of *Acropora* and *Pocillopora*. The timescales over which this recovery occurred varied between sites, but recovery of coral cover and communities to pre-1998 levels/states was generally evident by around 2009 to 2012, depending on location^22^,^36^,^37^. Given these past recovery trajectories and the fact that local populations of key reef building corals (mainly below 5–6 m water depth) have survived this event the potential for the reefs to recovery must, in theory at least, be good. Indeed, based on past recovery trajectories a best estimate might be that after a period of reef framework breakdown, that recovery might be feasible with ~10 years.

However, there will be three key factors that influence future recovery. The first, as outlined above, will be the frequency and magnitude of future SST warming events, and which are predicted to increase in the near future^15^,^16^,^38^. The second will be the occurrence of crown-of-thorns starfish (COTS) outbreaks, which impacted some reefs in the central region of the Maldives in 2015^22^, although very few COTS were observed at our study sites in either sampling period in 2016. Third, will be the impacts of increasing direct anthropogenic stressors across the region. These include the impacts associated with further rapid expansion of resorts and any associated increases in sedimentation or eutrophication stress, and fishing pressures, and which may impede natural trajectories of coral community recover from, or resilience to, climate-driven stressors. For the Maldives in particular, any factors that result in long-term or more frequent suppression of shallow fore-reef carbonate budgets and reef growth potential may have profound implications because of the close spatial proximity between the reefs and the adjacent low-lying reef islands that define the archipelago. In particular, any reduction in reef structure and loss of growth potential will have likely implications for wave energy propagation across the lagoons, and for the capacity of these reefs to track IPCC projections of future sea level rise, both factors that will threaten shoreline stability.

## Methods

Surveys were conducted during January and September 2016 on five atoll interior reefs in the southern Maldivian atoll of Gaafu Dhaalu; Mahutigala, Kandahalagala, Kafigahlaa, Kodehutigalaa and Kadumaigala. All surveys were conducted along the fore-reef slope 2 m depth contour on the south-western margins of each reef. At each site 5 replicate survey lines (10 m long) were established running parallel to the reef crest, with a spacing of 5 m between transects. To quantify substrate composition, reef rugosity, and gross carbonate production and erosion, and thus to determine net carbonate budgets (G, where G = kg CaCO_3_ m^2^ yr^−1^) we used a previously modified version of the ReefBudget methodology^13^, as described in Perry *et al*.^23^. These metrics are calculated as a function of the 3-dimensional surface of the reefs. For the benthic assessments we measured the distance within each linear 1 m covered by each category of benthic cover beneath a 10 m guide line using a separate flexible tape. All overhangs, vertical surfaces and horizontal surfaces below the line were surveyed, with the following groups recorded: scleractinian corals to the genera and morphological level e.g., *Acropora* branching, *Porites* massive etc. (see Supplementary Table 3 for a summary of coral cover data); crustose coralline algae (CCA) including CCA below macroalgal or soft coral cover; turf algae; fleshy macroalgae; non encrusting coralline algae (e.g., *Halimeda* spp., articulated coralline algae); sediment; bare substrate (e.g., limestone pavement); sediment; rubble; and other benthic organisms. Substrate rugosity was calculated as total reef surface divided by linear distance (a completely flat surface would therefore have a rugosity of ref. 1).

Following the modified version of the ReefBudget approach described by Perry *et al*.^23^, we then used the morphology and size of individual coral colonies in combination with genera specific skeletal density (g cm^−3^) and linear growth rates (cm year^−1^) across each transect to estimates carbonate production rates in kg CaCO_3_ m^−2^ year^−1^. In most cases we used mean regional growth rates and densities for each coral genera (see Supplementary Table 4 for a summary of rates used). These data were then combined with geometric transformations based on colony morphology to give a growth rate for each colony for the area under the transect line (taking a transect line width of 1 cm): massive colonies were assumed to be hemispherical in cross-section; encrusting, foliose and plating colonies, as well as colonies of crustose coralline algae (CCA) grow primarily from their margins such that the rate of radial expansion decreases rapidly with increasing colony size^39^. To accommodate for this we thus make an assumption that the growth rate across the internal (older) portion of such colonies is much lower – factored here at a rate of 10% the rate of marginal expansion to account for secondary skeletal infilling; for branching colonies, the proportion of the colony area of growing branch tips was assumed to be growing at published rates, and the remainder of the colony at 10% of these rates (see ref. 23).

To calculate the production for a single transect over a year, the following equation was then used:


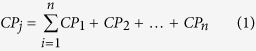


where *CP*_*j*_ is the total carbonate production of both corals and crustose coralline algae for transect *j* in kg CaCO_3_ yr^−1^. To estimate the production rate of the reef, we then used the following equation:


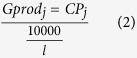


where *Gprod*_*j*_ is the carbonate production rate of both corals and crustose coralline algae for transect *j* in kg CaCO_3_ m^−2^ year^−1^, and l is the transect length in centimetres.

To quantify rates of biological substrate erosion we used three approaches for different elements of the bioeroding communities. Rates of macro- and microendolithic bioerosion were based on published rates per unit area derived from experimental studies in the Indo-Pacific region, and these were applied to all available dead carbonate substrate available to bioeroding sponges, including that covered by macroalgae or algal turf and live coral cover and soft corals^23^. To quantify echinoid bioerosion rates, a census of urchin abundance, size and species composition within 10 × 2 m belt transects was conducted along each benthic transect line. Typically these data would then be applied to published relationships between urchin species, test size and erosion rate, but no urchins were encountered during our surveys and they are thus assumed to exert little or no influence on carbonate erosion rates. Finally, to calculate bioerosion by parrotfish the species-size-life phase abundances of bioeroding parrotfish at each site were calculated at each site based on eight 30 m × 4 m belt transects, with all surveys completed by the same experienced observer (K.M.M.). Biomass of individual fish was then calculated using estimated length data and length-weight relationships and multiplied by abundance of the species or family of the fish (see ref. 23). To calculate parrotfish bioerosion rates by each individual fish we then used a model based on total length and life phase to predict the bite rates (bites hr^−1^) based on published data for that species or for similar sized species with the same feeding functional group. We then used the following equation to calculate species specific erosion rates for the median value within each size class:


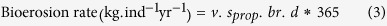


where *v* is bite volume (cm^3^), *s*_*prop*_ is the proportion of bites leaving scars, *b*_*r*_ is bite rate (bites day^−1^) and *d* is substratum density (kg cm^−3^), here taken to be 1.49 after Morgan & Kench (2012).

To assess changes in the accretion potential of reefs (mm yr^−1^) pre- and post-bleaching we converted our net production rate estimates to potential accretion rates (mm yr^−1^), using an approach previously applied to other Indian Ocean and Caribbean reefs^12^,^23^. Specifically, we estimated the maximum accretion potential of each reef as a function of the net carbonate production rate of the site (calculated as gross production less gross erosion rate) and assumed that a proportion of the bioeroded framework (that is converted to sediment) is also reincorporated back into the accumulating reef structure. This proportion is calculated as the sum of 50% of the parrotfish-derived sediment (as a highly mobile bioeroder which defecates randomly over the reef), as well as any sediment produced by urchins and by macroborer erosion. To keep our estimates conservative we worked on the assumption that only 50% of this bioerosional sediment yield is actually incorporated back into the reef (based on data in ref. 40), and excluded any sediment generation by other benthic sediment producers. Finally, we made an allowance for variations in the porosity of the accumulating reef framework as follows: 30% for head and massive coral dominated assemblages, 70% for branched and tabular dominated assemblages, and 50% for mixed coral assemblages (based on data in ref. 41). A loss factor to account for natural framework removal through physical processes was also included based on framework production and removal rates calculated from atoll interior reefs in the Maldives^42^,^43^, such that we assumed that 20% of the annual framework produced was removed from these relatively sheltered reef settings. T-tests were then used to test for significance of difference between net and gross production, erosion and accretion rates pre- and post-bleaching at each site.

## Additional Information

**How to cite this article**: Perry, C. T. and Morgan, K. M. Bleaching drives collapse in reef carbonate budgets and reef growth potential on southern Maldives reefs. *Sci. Rep.* **7**, 40581; doi: 10.1038/srep40581 (2017).

**Publisher's note:** Springer Nature remains neutral with regard to jurisdictional claims in published maps and institutional affiliations.

## Supplementary Material

Supplementary Tables
