# Supplementary material for: Bleaching drives collapse in reef carbonate budgets and reef growth potential on southern Maldives reefs
Source: Sci Rep. 2017 Jan 13;7:40581. doi: 10.1038/srep40581 (PMC5233991; doi:10.1038/srep40581)
Supplement: Supplementary Tables [file srep40581-s1.doc]

**Bleaching drives collapse in reef carbonate budgets and reef growth potential on southern Maldives reefs**

C.T. Perry & K.M. Morgan

**Supplementary Tables**

**Supplementary Table 1.** Summary of key ecological and budget metrics at sites in January 2016 and September 2016.

**Supplementary Table 2.** Results of paired t-tests comparing key ecological, structural and carbonate budget metrics between January 2016 and September 2016.

**Table SI 3.** Summary of coral cover data by genera for the five sites in (A) January 2016, and (B) September 2016.

**SI Table 4.** Mean extension and density rates (and sources) used in the coral carbonate production rate calculation methodology.

**Supplementary Table 1.** Summary of key ecological and budget metrics at sites in January 2016 and September 2016. All data were collected along transects running south-eastwards from the following GPS coordinates: Mahutigala (N00° 17' 20.3˝, E073° 11' 56.9˝); Kodehutigalaa (N00° 17' 29.8˝, E073° 11' 20.9˝); Kandahalaga (N00° 13' 33.3˝, E073° 12' 45.1˝); Kadumaigala (N00° 16' 55.0˝, E073° 10' 13.9˝); Kafigahlaa (N00° 17' 13.4˝, E073° 13' 14.9˝).

| Site | Site code | Survey date | Depth (m) | Coral production (G) | CCA production (G) | Parrotfish (G) | Urchin (G) | Macro- endolith bioerosion (G) | Micro-endolith bioerosion (G) | % coral contribution to total G | % of erosion by parrotfish | **NET G** | Coral cover % | Rugosity |
| --- | --- | --- | --- | --- | --- | --- | --- | --- | --- | --- | --- | --- | --- | --- |
| **JANUARY 2016** | | | | | | | | | | | | | | |
| Mahutigala, SW side | MAH-1 | 26/01/2016 | 2m | 7.25 ± 2.39 | 0.46±0.23 | 1.65±1.12 | 0.000 | 0.64±0.12 | 0.68±0.09 | 94.034 | 55.56 | **4.74 ±2.29** | 23.5 ±7.32 | 2.8±0.3 |
| Kodehutigalaa, SW side | KOD-1 | 28/01/2016 | 2m | 6.49± 0.53 | 0.55±0.13 | 2.11±1.38 | 0.000 | 0.61±0.06 | 0.64±0.05 | 92.188 | 62.81 | **3.68 ±0.30** | 20.7 ±2.93 | 2.6±0.2 |
| Kandahalaga, SW side | KAN-1 | 29/01/2016 | 2m | 11.30±4.28 | 0.29±0.12 | 1.76±1.28 | 0.000 | 0.57±0.16 | 0.59±0.11 | 97.498 | 60.27 | **8.67 ±4.16** | 34.9 ±7.99 | 2.5±0.4 |
| Kadumaigala, SW side | KAD-1 | 01/02/2016 | 2m | 7.14±2,31 | 0.54±0.15 | 1.79±1.12 | 0.000 | 0.61±0.12 | 0.63±0.05 | 92.953 | 59.08 | **4.65 ±2.26** | 21.4 ±5.85 | 2.5±0.2 |
| Kafigahlaa SW side | KAF-1 | 02/02/2016 | 2m | 9.95±3.09 | 0.62±0.16 | 1.46±1.23 | 0.000 | 0.59±0.13 | 0.64±0.09 | 94.144 | 54.14 | **7.87 ±3.09** | 27.2 ±7.96 | 2.6±0.3 |
| **SEPTEMBER 2016** | | | | | | | | | | | | | | |
| Mahutigala, SW side | MAH-1 | 16/09/2016 | 2m | 1.54±0.43 | 0.48±0.08 | 3.81±4.17 | 0.000 | 0.60±0.08 | 0.59±0.08 | 76.087 | 76.00 | **-2.99 ±0.40** | 5.26 ±2.11 | 2.5±0.3 |
| Kodehutigalaa, SW side | KOD-1 | 17/09/2016 | 2m | 1.63±1.00 | 0.55±0.06 | 3.42±2.26 | 0.000 | 0.59±0.04 | 0.59±0.04 | 74.840 | 74.23 | **-2.45 ±1.04** | 5.59 ±3.26 | 2.4±0.2 |
| Kandahalaga, SW side | KAN-1 | 15/09/2016 | 2m | 2.67±1.38 | 0.29±0.16 | 3.95±2.81 | 0.000 | 0.49±0.04 | 0.49±0.04 | 90.125 | 80.07 | **-1.97 ±1.39** | 9.78 ±3.8 | 2.2±0.2 |
| Kadumaigala, SW side | KAD-1 | 18/09/2016 | 2m | 1.64±1.05 | 0.58±0.33 | 5.89±5.92 | 0.000 | 0.54±0.15 | 0.53±0.17 | 73.940 | 84.67 | **-4.74 ±2.26** | 5.20 ±3.26 | 2.2±0.1 |
| Kafigahlaa SW side | KAF-1 | 19/06/2016 | 2m | 1.67±0.93 | 0.54±0.31 | 3.69±3.24 | 0.000 | 0.61±0.08 | 0.60±0.08 | 75.529 | 75.37 | **-2.68 ±0.94** | 5.53±3.08 | 2.3±0.3 |

**Supplementary Table 2.** Results of paired t-tests comparing key ecological, structural and carbonate budget metrics between January 2016 and September 2016.

Results of paired t-tests comparing mean coral cover (%) between January 2016 and September 2016.

| **Variable** | **January 2016** | | **September 2016** | |  | | |
| --- | --- | --- | --- | --- | --- | --- | --- |
|  | **Mean** | **SD** | **Mean** | **SD** | ***t(4)*** | ***p*** | **95% Cl** |
| Coral cover - Mahutigala | 23.46 | 7.32 | 5.26 | 2.11 | 5.0628 | 0.0072 | 8.21, 28.11 |
| Coral cover - Kodehutigalaa | 20.73 | 2.93 | 5.59 | 3.26 | 5.5330 | 0.0052 | 7.54, 22.74 |
| Coral cover - Kandahalagala | 34.91 | 7.99 | 9.78 | 3.86 | 5.6371 | 0.0049 | 12.75, 37.51 |
| Coral cover - Kadumaigala | 21.43 | 5.85 | 5.20 | 3.26 | 5.5519 | 0.0053 | 8.06, 24.39 |
| Coral cover - Kafigahlaa | 27.28 | 7.96 | 5.26 | 2.11 | 7.9347 | 0.0014 | 14.14, 29.35 |

Results of paired t-tests comparing mean substrate rugosity between January 2016 and September 2016.

| **Variable** | **January 2016** | | **September 2016** | |  | | |
| --- | --- | --- | --- | --- | --- | --- | --- |
|  | **Mean** | **SD** | **Mean** | **SD** | ***t(4)*** | ***p*** | **95% Cl** |
| Rugosity - Mahutigala | 2.80 | 0.30 | 2.48 | 0.29 | 1.5608 | 0.1939 | -0.25, 0.90 |
| Rugosity – Kodehutigalaa | 2.63 | 0.20 | 2.42 | 0.15 | 2.0937 | 0.1044 | -0.06, 0.48 |
| Rugosity – Kandahalagala | 2.47 | 0.39 | 2.18 | 0.22 | 1.8637 | 0.1358 | -0.14, 0.73 |
| Rugosity – Kadumaigala | 2.54 | 0.21 | 2.25 | 0.12 | 3.3907 | 0.0270 | 0.05, 0.54 |
| Rugosity – Kafigahlaa | 2.55 | 0.32 | 2.35 | 0.30 | 4.4143 | 0.0116 | 0.07, 0.33 |

Results of paired t-tests comparing mean carbonate budgets (G) between January 2016 and September 2016.

| **Variable** | **January 2016** | | **September 2016** | |  | | |
| --- | --- | --- | --- | --- | --- | --- | --- |
|  | **Mean** | **SD** | **Mean** | **SD** | ***t(4)*** | ***p*** | **95% Cl** |
| Net budget – Mahutigala | 4.73 | 2.28 | -2.98 | 0.40 | 6.8413 | 0.0024 | 4.58, 10.85 |
| Net budget – Kodehutigalaa | 3.63 | 0.30 | -2.42 | 1.04 | 11.1506 | 0.0004 | 4.55, 7.57 |
| Net budget – Kandahalagala | 8.66 | 4.15 | -1.96 | 1.38 | 4.9554 | 0.0077 | 4.67, 16.59 |
| Net budget – Kadumaigala | 4.65 | 2.26 | -4.73 | 0.93 | 8.5043 | 0.0010 | 6.32, 12.45 |
| Net budget – Kafigahlaa | 7.86 | 3.09 | -2.67 | 0.94 | 9.1535 | 0.0008 | 7.34, 13.74 |

Results of paired t-tests comparing mean coral carbonate production (G) between January 2016 and September 2016.

| **Variable** | **January 2016** | | **September 2016** | |  | | |
| --- | --- | --- | --- | --- | --- | --- | --- |
|  | **Mean** | **SD** | **Mean** | **SD** | ***t(4)*** | ***p*** | **95% Cl** |
| Coral G – Mahutigala | 7.25 | 2.43 | 1.54 | 0.43 | 4.6672 | 0.0095 | 2.31, 9.11 |
| Coral G – Kodehutigalaa | 6.48 | 0.26 | 1.63 | 1.00 | 9.7075 | 0.0006 | 3.46, 6.24 |
| Coral G – Kandahalagala | 11.31 | 4.28 | 2.67 | 1.38 | 3.9616 | 0.0167 | 2.58, 14.68 |
| Coral G – Kadumaigala | 7.15 | 2.30 | 1.64 | 0.93 | 4.7816 | 0.0088 | 2.31, 8.71 |
| Coral G – Kafigahlaa | 9.95 | 3.09 | 1.67 | 0.93 | 7.2322 | 0.0019 | 5.09, 11.45 |

Results of paired t-tests comparing mean parrotfish bioerosion (G) between January 2016 and September 2016.

| **Variable** | **January 2016** | | **September 2016** | |  | | |
| --- | --- | --- | --- | --- | --- | --- | --- |
|  | **Mean** | **SD** | **Mean** | **SD** | ***t(7)*** | ***p*** | **95% Cl** |
| Parrotfish G – Mahutigala | 1.65 | 1.12 | 3.81 | 4.17 | 2.6726 | 0.0319 | -4.14, -0.25 |
| Parrotfish G – Kodehutigalaa | 2.11 | 1.38 | 3.42 | 2.26 | 3.9140 | 0.0058 | -2.63, -0.64 |
| Parrotfish G – Kandahalagala | 1.76 | 1.28 | 3.95 | 2.81 | 3.1074 | 0.0171 | -4.82, -0.65 |
| Parrotfish G – Kadumaigala | 1.79 | 1.12 | 5.88 | 5.92 | 2.5915 | 0.0359 | -9.79, -0.44 |
| Parrotfish G – Kafigahlaa | 1.46 | 1.23 | 3.69 | 4.15 | 2.4009 | 0.0474 | -5.54, -0.04 |

Results of paired t-tests comparing % *Acropora* sp. between January 2016 and September 2016.

| **Variable** | **January 2016** | | **September 2016** | |  | | |
| --- | --- | --- | --- | --- | --- | --- | --- |
|  | **Mean** | **SD** | **Mean** | **SD** | ***t(4)*** | ***p*** | **95% Cl** |
| Acropora % – Mahutigala | 11.04 | 5.11 | 0.45 | 0.41 | 4.6129 | 0.0017 | 5.29, 15.88 |
| Acropora % – Kodehutigalaa | 11.57 | 2.99 | 1.56 | 2.72 | 5.5241 | 0.0006 | 5.82, 14.18 |
| Acropora % – Kandahalagala | 22.52 | 1.32 | 1.49 | 0.83 | 29.9757 | 0.0001 | 19.41, 22.65 |
| Acropora % – Kadumaigala | 11.34 | 4.90 | 1.03 | 1.12 | 4.5787 | 0.0018 | 5.11, 15.50 |
| Acropora % – Kafigahlaa | 17.14 | 3.91 | 1.43 | 1.05 | 8.6827 | 0.0001 | 11.54, 19.88 |

Results of paired t-tests comparing proportional contributions of *Acropora* sp. to coral carbonate production (G) between January 2016 and September 2016.

| **Variable** | **January 2016** | | **September 2016** | |  | | |
| --- | --- | --- | --- | --- | --- | --- | --- |
|  | **Mean** | **SD** | **Mean** | **SD** | ***t(4)*** | ***p*** | **95% Cl** |
| *Acropora* sp. – Mahutigala | 53.0 | 18.6 | 13.0 | 12.7 | 3.9587 | 0.0042 | 0.16, 0.63 |
| *Acropora* sp. – Kodehutigalaa | 59.4 | 9.63 | 17.0 | 19.1 | 4.4239 | 0.0022 | 0.20, 0.64 |
| *Acropora* sp. – Kandahalagala | 65.2 | 13.7 | 16.6 | 9.7 | 6.4372 | 0.0002 | 0.31, 0.66 |
| *Acropora* sp. – Kadumaigala | 52.0 | 15.2 | 14.6 | 12.7 | 4.2089 | 0.0030 | 0.16, 0.57 |
| *Acropora* sp. – Kafigahlaa | 56.8 | 11.1 | 22.0 | 7.1 | 5.6874 | 0.0005 | 0.20, 0.48 |

Results of paired t-tests comparing proportional contributions of massive and sub-massive taxa to coral carbonate production (G) between January 2016 and September 2016.

| **Variable** | **January 2016** | | **September 2016** | |  | | |
| --- | --- | --- | --- | --- | --- | --- | --- |
|  | **Mean** | **SD** | **Mean** | **SD** | ***t(4)*** | ***p*** | **95% Cl** |
| Massives – Mahutigala | 31.6 | 19.2 | 45.0 | 29.8 | 0.8445 | 0.4229 | -0.49, 0.23 |
| Massives – Kodehutigalaa | 10.4 | 6.1 | 29.4 | 24.9 | 1.6524 | 0.1371 | -0.45, 0.07 |
| Massives – Kandahalagala | 28.4 | 1.45 | 60.2 | 14.7 | 3.4371 | 0.0089 | -0.53, -0.10 |
| Massives – Kadumaigala | 22.8 | 20.2 | 58.6 | 26.7 | 2.3835 | 0.0443 | -0.70, -0.01 |
| Massives – Kafigahlaa | 5.4 | 4.9 | 45.0 | 37.7 | 2.3284 | 0.0483 | -0.78, 0.00 |

Results of paired t-tests comparing proportional contributions of non-*Acropora* branching taxa to coral carbonate production (G) between January 2016 and September 2016.

| **Variable** | **January 2016** | | **September 2016** | |  | | |
| --- | --- | --- | --- | --- | --- | --- | --- |
|  | **Mean** | **SD** | **Mean** | **SD** | ***t(4)*** | ***p*** | **95% Cl** |
| Other branch – Mahutigala | 1.6 | 3.1 | 15.0 | 19.6 | 1.5051 | 0.1707 | -0.33, 0.07 |
| Other branch – Kodehutigalaa | 11.6 | 10.9 | 28.8 | 11.1 | 2.4622 | 0.0392 | -0.33, -0.01 |
| Other branch – Kandahalagala | 2.0 | 2.9 | 9.2 | 10.5 | 1.4746 | 0.1785 | -0.18, 0.04 |
| Other branch – Kadumaigala | 3.4 | 5.6 | 3.8 | 6.1 | 0.1077 | 0.9169 | -0.08, 0.08 |
| Other branch – Kafigahlaa | 4.0 | 3.1 | 4.4 | 3.5 | 0.1894 | 0.8545 | -0.05, 0.04 |

Results of paired t-tests comparing proportional contributions of encrusting taxa to coral carbonate production (G) between January 2016 and September 2016.

| **Variable** | **January 2016** | | **September 2016** | |  | | |
| --- | --- | --- | --- | --- | --- | --- | --- |
|  | **Mean** | **SD** | **Mean** | **SD** | ***t(4)*** | ***p*** | **95% Cl** |
| Encrusting – Mahutigala | 10.0 | 5.1 | 14.8 | 10.4 | 0.9231 | 0.3830 | -0.16, 0.07 |
| Encrusting – Kodehutigalaa | 7.0 | 4.1 | 18.6 | 24.7 | 1.0348 | 0.3310 | -0.37, 0.14 |
| Encrusting – Kandahalagala | 3.0 | 1.8 | 8.8 | 5.7 | 2.1408 | 0.0647 | -0.12, 0.01 |
| Encrusting – Kadumaigala | 4.6 | 4.2 | 4.2 | 4.0 | 0.1523 | 0.8827 | -0.05, 0.06 |
| Encrusting – Kafigahlaa | 3.4 | 1.3 | 3.6 | 4.5 | 0.0951 | 0.9266 | -0.05, 0.04 |

Results of paired t-tests comparing calculated reef accretion rates (mm^-1^) between January 2016 and September 2016.

| **Variable** | **January 2016** | | **September 2016** | |  | | |
| --- | --- | --- | --- | --- | --- | --- | --- |
|  | **Mean** | **SD** | **Mean** | **SD** | ***t(4)*** | ***p*** | **95% Cl** |
| Accretion – Mahutigala | 3.55 | 1.34 | -0.45 | 0.25 | 6.5245 | 0.0002 | 2.59, 5.42 |
| Accretion – Kodehutigalaa | 2.99 | 0.14 | -0.22 | 0.55 | 12.5367 | 0.0001 | 2.62, 3.80 |
| Accretion – Kandahalagala | 5.69 | 2.41 | -0.03 | 0.76 | 5.0665 | 0.0010 | 3.11, 8.32 |
| Accretion – Kadumaigala | 3.48 | 1.24 | -1.14 | 0.48 | 7.7083 | 0.0001 | 3.24, 6.01 |
| Accretion – Kafigahlaa | 5.23 | 1.72 | -0.31 | 0.50 | 6.9017 | 0.0001 | 3.68, 7.38 |

**Table SI 3.** Summary of coral cover data by genera for the five sites in (A) January 2016, and (B) September 2016. NB. Percent cover data is measured and reported as the total of the true 3-dimensional surface of the reef and hence are lower than rates that would be measured using standard linear point or video methodologies.

A) Summary of coral cover data by genera for the five sites in January 2016.

|  | **Mahutigala - Jan 2016** | | **Kandahalagala - Jan 2016** | | **Kodehutigala - Jan 2016** | | **Kadumaigala - Jan 2016** | | **Kafigalaa - Jan 2016** | |
| --- | --- | --- | --- | --- | --- | --- | --- | --- | --- | --- |
| **Genera** | **Mean %** | **SD** | **Mean %** | **SD** | **Mean %** | **SD** | **Mean %** | **SD** | **Mean %** | **SD** |
| *Acropora* | 11.05 | 5.12 | 22.53 | 1.33 | 11.58 | 3.00 | 11.46 | 5.10 | 17.14 | 3.91 |
| *Astreopora* | 0.08 | 0.18 | 0.54 | 0.53 | 0.66 | 1.47 | 0.00 | 0.00 | 0.33 | 0.74 |
| *Coscinarea* | 0.00 | 0.00 | 0.00 | 0.00 | 0.00 | 0.00 | 0.00 | 0.00 | 0.00 | 0.00 |
| *Cyphastrea* | 0.57 | 1.28 | 0.00 | 0.00 | 0.00 | 0.00 | 0.49 | 0.70 | 0.00 | 0.00 |
| *Diploastrea* | 0.00 | 0.00 | 0.00 | 0.00 | 0.00 | 0.00 | 0.00 | 0.00 | 0.00 | 0.00 |
| *Echinopora* | 0.00 | 0.00 | 0.30 | 0.67 | 0.00 | 0.00 | 0.52 | 0.88 | 0.13 | 0.29 |
| *Favia* | 0.68 | 0.94 | 1.50 | 1.43 | 0.09 | 0.20 | 0.06 | 0.13 | 0.62 | 1.05 |
| *Favites* | 0.00 | 0.00 | 0.00 | 0.00 | 0.00 | 0.00 | 0.51 | 1.15 | 0.27 | 0.41 |
| *Fungia* | 0.51 | 0.71 | 0.19 | 0.28 | 0.63 | 0.39 | 1.92 | 1.06 | 3.46 | 2.25 |
| *Galaxea* | 0.00 | 0.00 | 0.37 | 0.58 | 0.15 | 0.35 | 0.00 | 0.00 | 0.18 | 0.40 |
| *Goniastrea* | 0.49 | 1.09 | 1.10 | 1.59 | 0.12 | 0.17 | 0.00 | 0.00 | 0.00 | 0.00 |
| *Goniopora* | 0.00 | 0.00 | 0.37 | 0.52 | 0.00 | 0.00 | 0.00 | 0.00 | 0.00 | 0.00 |
| *Unidentified Hard coral* | 0.00 | 0.00 | 0.00 | 0.00 | 1.21 | 1.65 | 0.00 | 0.00 | 0.00 | 0.00 |
| *Halomitra* | 0.05 | 0.11 | 0.00 | 0.00 | 0.00 | 0.00 | 0.00 | 0.00 | 0.09 | 0.21 |
| *Herpolitha* | 0.00 | 0.00 | 0.06 | 0.13 | 0.11 | 0.25 | 0.48 | 0.66 | 1.56 | 1.50 |
| *Hydnophora* | 0.00 | 0.00 | 0.18 | 0.41 | 0.00 | 0.00 | 0.00 | 0.00 | 0.00 | 0.00 |
| *Leptastrea* | 0.40 | 0.43 | 0.16 | 0.37 | 0.39 | 0.61 | 0.00 | 0.00 | 0.00 | 0.00 |
| *Leptoseris* | 1.73 | 0.82 | 0.00 | 0.00 | 0.82 | 0.86 | 0.43 | 0.51 | 0.00 | 0.00 |
| *Lobophyllia* | 0.00 | 0.00 | 0.00 | 0.00 | 0.00 | 0.00 | 0.00 | 0.00 | 0.00 | 0.00 |
| *Millepora* | 0.00 | 0.00 | 0.00 | 0.00 | 0.00 | 0.00 | 0.00 | 0.00 | 0.00 | 0.00 |
| *Montastrea* | 1.25 | 1.82 | 0.44 | 0.45 | 0.87 | 1.08 | 0.51 | 0.37 | 0.07 | 0.17 |
| *Pachyseris* | 0.00 | 0.00 | 0.00 | 0.00 | 0.32 | 0.31 | 0.00 | 0.00 | 0.00 | 0.00 |
| *Pavona* | 0.33 | 0.73 | 0.00 | 0.00 | 0.00 | 0.00 | 0.13 | 0.30 | 0.00 | 0.00 |
| *Pocillopora* | 0.21 | 0.36 | 0.48 | 0.81 | 1.67 | 1.56 | 0.58 | 0.92 | 0.69 | 0.65 |
| *Porites* | 5.75 | 4.23 | 5.59 | 4.33 | 1.37 | 0.88 | 3.76 | 3.62 | 0.68 | 0.76 |
| *Psammacora* | 0.34 | 0.76 | 0.88 | 0.92 | 0.41 | 0.41 | 0.57 | 0.42 | 1.20 | 1.46 |
| *Seriatopera* | 0.00 | 0.00 | 0.00 | 0.00 | 0.00 | 0.00 | 0.00 | 0.00 | 0.00 | 0.00 |
| *Stylophora* | 0.00 | 0.00 | 0.05 | 0.10 | 0.00 | 0.00 | 0.00 | 0.00 | 0.27 | 0.36 |
| *Symphillia* | 0.00 | 0.00 | 0.00 | 0.00 | 0.33 | 0.73 | 0.00 | 0.00 | 0.07 | 0.15 |

B) Summary of coral cover data by genera for the five sites in September 2016.

|  | **Mahutigala - Sept 2016** | | **Kandahalagala - Sept 2016** | | **Kodehutigala - Sept 2016** | | **Kadumaigala - Sept 2016** | | **Kafigalaa - Sept 2016** | |
| --- | --- | --- | --- | --- | --- | --- | --- | --- | --- | --- |
| **Genera** | **Mean %** | **SD** | **Mean %** | **SD** | **Mean %** | **SD** | **Mean %** | **SD** | **Mean %** | **SD** |
| *Acropora* | 0.45 | 0.42 | 1.50 | 0.84 | 1.57 | 2.73 | 1.04 | 1.13 | 1.43 | 1.06 |
| *Astreopora* | 0.07 | 0.15 | 0.18 | 0.25 | 0.23 | 0.51 | 0.00 | 0.00 | 0.00 | 0.00 |
| *Coscinarea* | 0.00 | 0.00 | 0.22 | 0.32 | 0.00 | 0.00 | 0.00 | 0.00 | 0.00 | 0.00 |
| *Cyphastrea* | 0.10 | 0.22 | 0.15 | 0.34 | 0.12 | 0.27 | 0.00 | 0.00 | 0.00 | 0.00 |
| *Diploastrea* | 0.00 | 0.00 | 0.00 | 0.00 | 0.00 | 0.00 | 0.00 | 0.00 | 0.06 | 0.14 |
| *Echinopora* | 0.00 | 0.00 | 0.08 | 0.17 | 0.00 | 0.00 | 0.10 | 0.23 | 0.00 | 0.00 |
| *Favia* | 0.21 | 0.47 | 0.85 | 0.81 | 0.04 | 0.10 | 0.00 | 0.00 | 0.04 | 0.09 |
| *Favites* | 0.00 | 0.00 | 0.89 | 0.88 | 0.00 | 0.00 | 0.00 | 0.00 | 0.42 | 0.69 |
| *Fungia* | 0.52 | 0.68 | 0.37 | 0.57 | 0.44 | 0.61 | 1.40 | 1.32 | 1.18 | 1.52 |
| *Galaxea* | 0.00 | 0.00 | 0.47 | 0.60 | 0.00 | 0.00 | 0.00 | 0.00 | 0.13 | 0.30 |
| *Goniastrea* | 0.70 | 0.84 | 0.00 | 0.00 | 0.23 | 0.51 | 0.33 | 0.74 | 0.25 | 0.56 |
| *Goniopora* | 0.00 | 0.00 | 0.00 | 0.00 | 0.00 | 0.00 | 0.00 | 0.00 | 0.00 | 0.00 |
| *Unidentified HC* | 0.00 | 0.00 | 0.00 | 0.00 | 0.00 | 0.00 | 0.00 | 0.00 | 0.00 | 0.00 |
| *Halomitra* | 0.00 | 0.00 | 0.00 | 0.00 | 0.00 | 0.00 | 0.00 | 0.00 | 0.00 | 0.00 |
| *Herpolitha* | 0.00 | 0.00 | 0.00 | 0.00 | 0.00 | 0.00 | 0.00 | 0.00 | 0.00 | 0.00 |
| *Hydnophora* | 0.00 | 0.00 | 0.00 | 0.00 | 0.20 | 0.44 | 0.00 | 0.00 | 0.00 | 0.00 |
| *Leptastrea* | 0.15 | 0.33 | 0.00 | 0.00 | 0.15 | 0.34 | 0.21 | 0.46 | 0.00 | 0.00 |
| *Leptoseris* | 1.54 | 2.16 | 0.43 | 0.32 | 0.53 | 0.70 | 0.10 | 0.21 | 0.00 | 0.00 |
| *Lobophyllia* | 0.00 | 0.00 | 0.00 | 0.00 | 0.00 | 0.00 | 0.16 | 0.36 | 0.00 | 0.00 |
| *Millepora* | 0.00 | 0.00 | 0.00 | 0.00 | 0.00 | 0.00 | 0.00 | 0.00 | 0.00 | 0.00 |
| *Montastrea* | 0.00 | 0.00 | 0.20 | 0.45 | 0.00 | 0.00 | 0.00 | 0.00 | 0.00 | 0.00 |
| *Pachyseris* | 0.00 | 0.00 | 0.00 | 0.00 | 0.12 | 0.28 | 0.31 | 0.69 | 0.00 | 0.00 |
| *Pavona* | 0.00 | 0.00 | 0.00 | 0.00 | 0.00 | 0.00 | 0.00 | 0.00 | 0.57 | 1.27 |
| *Pocillopora* | 0.44 | 0.50 | 0.53 | 0.51 | 1.02 | 0.37 | 0.00 | 0.00 | 0.00 | 0.00 |
| *Porites* | 0.61 | 0.88 | 3.93 | 3.47 | 0.93 | 0.77 | 1.56 | 1.37 | 1.35 | 1.18 |
| *Psammacora* | 0.36 | 0.52 | 0.00 | 0.00 | 0.00 | 0.00 | 0.00 | 0.00 | 0.00 | 0.00 |
| *Seriatopera* | 0.12 | 0.27 | 0.00 | 0.00 | 0.00 | 0.00 | 0.00 | 0.00 | 0.00 | 0.00 |
| *Stylophora* | 0.00 | 0.00 | 0.00 | 0.00 | 0.00 | 0.00 | 0.00 | 0.00 | 0.00 | 0.00 |
| *Symphillia* | 0.00 | 0.00 | 0.00 | 0.00 | 0.00 | 0.00 | 0.00 | 0.00 | 0.00 | 0.00 |

**SI Table 4.** Mean extension and density rates (and sources) used in the coral carbonate production rate calculation methodology.

| **Coral genera** | **Coral growth morphology** | **Mean Extension rate (cm yr^-1^)** | **Mean Density (g cm^3^)** | **Notes on sources/ substitutions** | **Source** |
| --- | --- | --- | --- | --- | --- |
| *Acropora* | arborescent | 6.931 | 1.294 | Used genera/morphology average | Browne (2012); Bucher et al. (1998); Charuchinda & Hylleberg (1984); Crossland (1981); Harriott, (1999); Morgan & Kench (2012); Neudecker (1981); Oliver et al. (1983);  Roche et al. (2010); Yap & Gomez (1985) |
| *Acropora* | branching | 4.154 | 1.340 | Used genera/morphology average | Bucher et al. (1998); Harriott (1999); Marsh (1993) Morgan & Kench (2012); Shi et al. (2008). |
| *Acropora* | corymbose | 4.282 | 1.340 | Used genera/morphology average | Bucher et al. (1998); Jokiel & Tyler (1992); Plucer-Rosario & Randall (1987); Shi et al. (2008). |
| *Acropora* | digitate | 2.987 | 1.227 | Used genera/morphology average | Brown et al. (1985); Bucher et al. (1998). Harriott (1999); Jokiel & Tyler (1992 |
| *Acropora* | table | 8.285 | 1.455 | Used genera/morphology average | Brown et al. (1985); Harriott (1999); Jokiel & Tyler (1992); Ma (1958); Stimson (1996) |
| *Astreopora* | encrusting, massive | 1.029 | 1.480 | Used genera/morphology average | Buddemeier et l. (1974); Shi et al. (2008) |
| *Coscinarea* | columnar, encrusting, massive | 1.785 | 1.405 | Used average for *Siderastredae* due to place in phylogentic tree | Buddemeier et al. (1974); Guzman & Cortes (1989); Roberts & Harriott (2003) |
| *Cyphastrea* | columnar, encrusting, massive | 0.532 | 1.358 | Used genera/morphology average | Harriott, (1999); Roberts & Harriott (2003); Shi et al. (2008) |
| *Diploastrea* | encrusting,  massive | 0.550 | 1.613 | Used genera/morphology average | Watanabe et al. (2003); Shi et al. (2008) |
| *Echinopora* | encrusting, foliose, massive | 0.821 | 1.358 | Used average for *Merulinidae*, *Faviidae* for density | Harriott (1999); Morgan & Kench (2012); Shi et al. (2008); Stimson (1996) |
| *Favia* | encrusting, massive | 0.624 | 1.345 | Used genera/morphology average | Buddemeier et al. (1979); Harriott (1999); Highsmith (1979); Shi et al. (2008) |
| *Favites* | encrusting, massive | 0.624* | 1.345* | Used genera/morphology average | Buddemeier et al. (1979); Harriott (1999); Highsmith (1979); Shi et al. (2008) |
| *Fungia* | mushroom | 0.730 | 1.990 | Used genera/morphology average | Jokiel & Tyler (1992); Ma (1958); Morgan & Kench (2012) |
| *Galaxea* | columnar, encrusting, massive | 0.900 | 1.910 |  | Shi et al. (2008) |
| *Goniastrea* | columnar, encrusting, massive | 1.200 | 1.726 | Used genera/morphology average | Anthony et al. (2002); Babcock (1988); Buddemeier et al. (1974); Harriott (1999); Roberts & Harriott (2003); Shi et al. (2008) |
| *Halomitra* | mushroom | 0.784 | 1.990 | Used average for mushroom corals | Buddemeier et al. (1974); Jokiel & Tyler (1992); Ma (1958); Morgan & Kench (2012) |
| *Hydnophora* | Massive, sub-massive | 1.110 | 1.370 | Used genera/morphology average | Buddemeier et al. (1974); Morgan & Kench (2012); Shi et al. (2008) |
| *Leptastrea* | encrusting, massive | 0.624 | 1.345 |  | Morgan & Kench (2012) |
| *Leptoria* | encrusting, massive | 0.821 | 1.358 | Used *Platygyra* averages | Babcock (1988); Buddemeier et al. (1974); Shi et al. (2008); Weber & White (1974) |
| Leptoseris | encrusting, foliose, plating | 1.669 | 1.726 |  | Plucer-Rosario & Randall (1987) |
| *Lobophyllia* | encrusting, massive | 1.500 | 1.370 |  | Shi et al. (2008) |
| *Millepora* | encrusting | 1.850 | 0.929 |  | Jokiel & Tyler (1992) |
| *Montastrea* | columnar, encrusting, massive | 0.260 | 1.358 | Used density average for *Faviidae* | Harriott (1999) |
| *Pachyseris* | encrusting | 1.199 | 1.726 | Use average for *Agariicidae* | Guzman & Cortes (1989); Jokiel & Tyler (1992); Manzello (2010); Plucer-Rosario & Randall (1987); Shi et al. (2008); Wellington (1982) |
| *Pavona* | columnar | 1.215 | 1.726 | Used genera/morphology average | Jokiel & Tyler (1992); Manzello (2010); Shi et al. (2008) |
| *Pavona* | encrusting | 0.335 | 1.726 | Used genera/morphology average | Guzman & Cortes (1989); Manzello (2010) |
| *Pavona* | foliose | 2.712 | 1.726 |  | Plucer-Rosario & Randall (1987) |
| *Pavona* | massive | 1.229 | 1.726 | Used genera/morphology average | Guzman & Cortes (1989); Manzello (2010); Plucer-Rosario & Randall (1987); Wellington (1982) |
| *Platygyra* | encrusting, massive | 0.874 | 1.527 | Used genera/morphology average | Babcock (1988); Buddemeier et al. (1974); Shi et al. (2008); Weber & White (1974) |
| *Pocillopora* | branching | 2.735 | 1.400 | Used genera/morphology average | Crossland (1981); Davies (1989); Glynn et al. (1979); Guzman & Cortes (1989); Harriott (1999); Jokiel & Tyler (1992); Morgan & Kench (2012); Neudecker (1981); Shi et al. (2008); Ward (1995); Wellington (1982) |
| *Pocillopora* | submassive | 3.536 | 1.400 | Used genera/morphology average | Buddemeier et al. (1979); Jokiel & Taylor (1992); Morgan & Kench (2012) |
| *Porites* | branching | 2.719 | 1.400 | Used genera/morphology average | Anthony et al. (2002); Cox (1986); Grottoli (1999); Morgan & Kench (2012); Neudecker (1981) |
| *Porites* | encrusting, massive | 1.169 | 1.372 | Used genera/morphology average | Bessat & Buigues (2001); Buddemeier et al. (1974); Cabral-Tena et al. (2013); Cantin & Lough (2014); Chen et al. (2013); Cooper et al. (2008); De’ath et al. (2009); Grottoli (1999); Grove et al. (2010); Guzmán & Cortés (1989); Harriott (1999); Highsmith (1979); Klein & Loya (1991); Lough & Barnes (1992); Merschel (2012); Morgan & Kench (2012); Shi et al. (2008); Smith et al. (2007). |
| *Psammacora* | encrusting, massive, plating | 1.785 | 1.405* | Used genera/morphology average | Buddemeier et al (1974); Guzman & Cortes (1989); Roberts & Harriott (2003) |
| *Seriatopora* | branching | 1.670 | 1.239 | Used density average from all coral genera | Harriott (1999) |
| *Stylophora* | branching | 2.550 | 1.405 |  | Klein & Loya (1991) |
| *Symphillia* | massive | 1.500 | 1.370 |  | Shi et al. (2008) |

**References**

Anthony, KRN, Connolly, SR, Willis, BL (2002). Comparative analysis of energy allocation to tissue and skeletal growth in corals. Limnology and Oceanography, 47: 1417–1429,

Babcock R (1988) Age-structure, survivorship and fecundity in populations of massive corals. Proc 6th Int Coral Reef Symposium, Townsville, Australia 2: 625-633.

Bessat, F & Buigues, D (2001) Two centuries of variation in coral growth in a massive *Porites* colony from Moorea (French Polynesia): a response of ocean-atmosphere variability from south. Palaeogeography, Palaeoclimatology, Palaeoecology, 175: 381–392.

Brown, B, Sya’Rani, L & Tissier, M Le (1985) Skeletal form and growth in *Acropora aspera* (Dana) from the Pulau Seribu, Indonesia. Journal of Experimental Marine Biology & Ecology, 86: 139–150.

Browne, NK (2012) Spatial and temporal variations in coral growth on an inshore turbid reef subjected to multiple disturbances. Marine Environmental Research, *77*: 71–83.

Bucher, DJ, Harriott, VJ & Roberts, LG (1998) Skeletal micro-density, porosity and bulk density of *Acroporid* corals. Journal of Experimental Marine Biology & Ecology, 228: 117–136.

Buddemeier R, Maragos J, Knutson D (1974) Radiographic studies of reef coral exoskeletons: rates and patterns of coral growth. J Experimental Marine Biology & Ecology, 14: 179-200.

Cabral-Tena, R, Reyes-Bonilla, H, Lluch-Cota, S, Paz-García, D, Calderón-Aguilera, L, Norzagaray-López, O & Balart, E (2013). Different calcification rates in males and females of the coral *Porites panamensis* in the Gulf of California. Marine Ecology Progress Series, 476: 1–8.

Cantin, NE & Lough, JM (2014). Surviving coral bleaching events: *Porites* growth anomalies on the Great Barrier Reef. PloS One, 9(2), e88720.

Charuchinda M, Hylleberg J (1984) Skeletal extension of *Acropora formosa* at a fringing reef in the Andaman Sea. Coral Reefs 17: 215–219.

Chen, T, Li, S, Yu, K, Zheng, Z, Wang, L & Chen, T (2013). Increasing temperature anomalies reduce coral growth in the Weizhou Island, northern South China Sea. Estuarine, Coastal and Shelf Science, 130: 121–126.

Cooper, TF, De’Ath, G, Fabricius, KE & Lough, JM (2008). Declining coral calcification in massive Porites in two nearshore regions of the northern Great Barrier Reef. Global Change Biology, 14: 529–538.

Cox E (1986) The effects of a selective corallivore on growth rates and competition for space between two species of Hawaiian corals. Journal of Experimental Marine Biology & Ecology, 101: 161–174.

Crossland C (1981) Seasonal growth of *Acropora cf. formosa* and *Pocillopora damicornis* on a high latitude reef (Houtman Abrolhos, Western Australia). Proc 4th Int Coral Reef Symposium, 1: 663–667.

Davies, P (1989). Short-term growth measurements of corals using an accurate buoyant weighing technique. Marine Biology, 395: 389–395.

De’ath, G, Lough, J & Fabricius, K (2009). Declining coral calcification on the Great Barrier Reef. Science, 323: 116–120.

Glynn P, Wellington G, Birkeland C (1979) Coral reef growth in the Galapagos: limitation by sea urchins. Science 80: 8–10.

Grottoli A. G (1999) Variability of stable isotopes and maximum linear extension in reef-coral skeletons at Kaneohe Bay, Hawaii. Marine Biology 135: 437–449.

Grove CA, Nagtegaal R, Zinke J, Sceufen T, Koster B, Kasper S, McCulloch MT, van den Bergh G, Brummer G Jan A (2010) River runoff reconstructions from novel spectral luminescence scanning of massive coral skeletons. Coral Reefs. 29: 579-591.

Guzmán H & Cortés J (1989) Growth rates of eight species of scleractinian corals in the eastern Pacific (Costa Rica). Bulletin Marine Science 44: 1186–1194.

Harriott, VJ (1999). Coral growth in subtropical eastern Australia. Coral Reefs, 18: 281–291.

Highsmith R (1979) Coral growth rates and environmental control of density banding. Journal of Experimental Marine Biology & Ecology, 37: 105–125.

Jokiel, P & Tyler, W (1992). Distribution of stony corals in Johnston Atoll lagoon. Proc. Seventh Int. Coral Reef Symposium, 2: 683–692.

Klein R, Loya Y (1991) Skeletal growth and density patterns of two *Porites* corals from the Gulf of Eilat, Red Sea. Mar Ecol Prog Ser Oldend 77: 253–259.

Lough, J & Barnes, D (1992). Comparisons of skeletal density variations in Porites from the central Great Barrier Reef. Journal of Experimental Marine Biology and Ecology, 155: 1–25.

Ma, TYH (1958) The relation of growth rate of reef corals to surface temperature of sea water as a basis for study of causes of disastrophisms instigating evolution of life. World Book Co, LTD, Taipei, Taiwan.

Manzello, DP (2010). Coral growth with thermal stress and ocean acidification: lessons from the eastern tropical Pacific. Coral Reefs, 29: 749–758.

Marsh L (1993) The occurrence and growth of *Acropora* in extra-tropical waters off Perth, Western Australia. Proc 7th Int Coral Reef Symposium, 2: 1233–1238.

Merschel G (2012) Reconstructing Past Climate Signals of northern Mozambique using Giant Corals. Masters thesis. VU University, Amseterdam.

Morgan KM & Kench PS (2012) Skeletal extension and calcification of reef-building corals in the central Indian Ocean. Marine Environmental Research. 81: 78-82.

Neudecker S (1981) Growth and survival of scleractinian corals exposed to thermal effluents at Guam. Proc 4th Int Coral Reef Symposium, 1:173–180

Oliver J, Chalker B, Dunlap W (1983) Bathymetric adaptations of reef-building corals at davies reef, great barrier reef, Australia. I. Long-term growth responses of *Acropora Formosa* Dana 1846). Journal of Experimental Marine Biology and Ecology, 73: 11–35.

Plucer-Rosario G, Randall R (1987) Preservation of rare coral species by transplantation and examination of their recruitment and growth. Bull Marine Science 41: 585–593.

Roberts LG, Harriott VJ (2003) Can environmental records be extracted from coral skeletons from Moreton Bay, Australia, a subtropical, turbid environment? Coral Reefs 22: 517–522.

Roche, RC, Abel, RA, Johnson, KG & Perry, CT (2010). Quantification of porosity in *Acropora pulchra* (Brook 1891) using X-ray micro-computed tomography techniques. Journal of Experimental Marine Biology and Ecology, 396: 1–9.

Shi, Q, Zhao, M, Zhang, Q, Yu, K, Chen, T, Li, S & Wang, H (2008). Estimate of carbonate production by scleractinian corals at Luhuitou fringing reef, Sanya, China. Chinese Science Bulletin, 54: 696–705.

Smith, LW, Barshis, D & Birkeland, C (2007). Phenotypic plasticity for skeletal growth, density and calcification of *Porites lobata* in response to habitat type. Coral Reefs, 26: 559–567.

Stimson J (1996) Wave like outward growth of some table- and plate- forming corals, and a hypothetical mechanism. Bulletin of Marine Science 58: 301–313.

Ward S (1995) The effect of damage on the growth, reproduction and storage of lipids in the scleractinian coral *Pocillopora damicornis* (Linnaeus). Journal of Experimental Marine Biology and Ecology 187: 193–206.

Watanabe T, Gagan MK, Correge T, Scott-Gagan H, Cowley J, Hantoro WS (2003) Oxygen isotope systematics in Diploastrea heliopora: New coral archive of tropical paleoclimate. Geochimica et Cosmochimica Acta 67: 1349-1358.

Weber J, White E (1974) Activation energy for skeletal aragonite deposited by the hermatypic coral *Platygyra* spp. Marie Biology, 359: 353–359

Wellington G (1982) An experimental analysis of the effects of light and zooplankton on coral zonation. Oecologia: 311–320.

Yap H, Gomez E (1985) Growth of *Acropora pulchra*. Marine Biology 87: 203–209.
